# Targeting of GLUT5 for Transporter-Mediated Drug-Delivery Is Contingent upon Substrate Hydrophilicity

**DOI:** 10.3390/ijms22105073

**Published:** 2021-05-11

**Authors:** Nazanin Nahrjou, Avik Ghosh, Marina Tanasova

**Affiliations:** 1 Chemistry Department, Michigan Technological University, Houghton, MI 49931, USA; nnrahjou@mtu.edu (N.N.); avikg@mtu.edu (A.G.); 2 Health Research Institute, Michigan Technological University, Houghton, MI 49931, USA

**Keywords:** sugar transport, fructose transport, GLUT5, drug conjugates, targeted delivery, cancer selectivity

## Abstract

Specific link between high fructose uptake and cancer development and progression highlighted fructose transporters as potential means to achieve GLUT-mediated discrimination between normal and cancer cells. The gained expression of fructose-specific transporter GLUT5 in various cancers offers a possibility for developing cancer-specific imaging and bioactive agents. Herein, we explore the feasibility of delivering a bioactive agent through cancer-relevant fructose-specific transporter GLUT5. We employed specific targeting of GLUT5 by 2,5-anhydro-D-mannitol and investigated several drug conjugates for their ability to induce cancer-specific cytotoxicity. The proof-of-concept analysis was carried out for conjugates of chlorambucil (CLB) in GLUT5-positive breast cancer cells and normal breast cells. The cytotoxicity of conjugates was assessed over 24 h and 48 h, and significant dependence between cancer-selectivity and conjugate size was observed. The differences were found to relate to the loss of GLUT5-mediated uptake upon increased conjugate size and hydrophobicity. The findings provide information on the substrate tolerance of GLUT5 and highlight the importance of maintaining appropriate hydrophilicity for GLUT-mediated delivery.

## 1. Introduction

The development of targeted approaches is the ultimate goal to achieve improvements in disease diagnostics and treatment. The chronic proliferation of cells representing the essence of neoplasia requires rapid consumption of nutrients compared to non-transformed tissues. Almost a century ago, the difference in the effectiveness of glycolysis and the citric acid cycle to produce energy or adapt to alteration in glycolysis efficiency (Warburg effect) was established as one of the characterizations that discriminate cancer cells from normal cells [1,2,3]. As a consequence of enhanced energy requirements, higher sugar concentrations are also needed for anabolic reactions to continue replication. Higher sugar uptake in cancers is reflected by the elevated activity and gained expression of facilitative sugar transporters—GLUTs. GLUTs are not coupled with energy, and sugar translocation across the cell membrane occurs via gradient-dependent influx and efflux of carbohydrates [4]. Individual GLUT isoforms demonstrate different tissue specificity, substrate specificity, and kinetic characteristics. Glucose is the predominant one among various carbohydrates that are transported by GLUTs. In addition to glucose, GLUTs supply cells with galactose, fructose, and other sugars. The transport kinetics and affinity for sugars differ between GLUTs, with the majority taking up more than one substrate and selected few showing substrate specificities [5,6].

The diversity of GLUTs allows for a tissue-specific adaptation of sugar uptake via regulation of gene expression [7]. The overall differences in GLUT composition between cells in conjunction with higher sugar consumption in cancer cells have provided a strong basis to view GLUTs as important therapeutic targets. Significant impact on advancing disease detection has been made by the development of the ^18^F-labeled analog of 2-fluoro-2-deoxy-D-glucose (2-^18^F-FDG) to target glucose-transporting GLUTs and its use in the clinic as a reflector of enhanced glucose uptake and predictor of tumorigenesis and tumor aggressiveness [8,9,10,11,12]. However, a large number of false-positive results [13] have highlighted the lack of required discrimination of cancer cells when targeting glucose GLUTs. Directing drugs or imaging agents to GLUTs is primarily done through corresponding glycoconjugates. Glucose conjugates of various bioactive or imaging agents have been synthesized and studied [14,15,16]. However, despite a large number of efforts, the examples of achieved improvements in toxicity and selectivity of bioactive agents due to the GLUT-mediated uptake are very limited. Limited is also the understanding of factors that contribute to a broadly observed loss of GLUT-mediated uptake upon derivatization of sugars.

The growing understanding of GLUTs-disease connections has identified several transporters that appear to play explicit roles in disease development and progression. Deregulation of nonspecific glucose and fructose transporter GLUT2 were found to be linked to obesity and diabetes [17], and high GLUT2 expression is viewed as a prognostic factor for liver cancers [18]. The glucose transporter GLUT3 gains expression in various types of cancer while primarily expressed strictly in the brain [7]. Fructose transporter GLUT5 is associated with various cancers, as well as obesity, fatty liver disease, and other metabolic deregulations [19]. The expression of nonspecific glucose and fructose transporter GLUT12 is established for early-stage and late-stage breast cancers [20]. While these direct links highlight the feasibility of impacting metabolically compromised cells directly through relevant nutrient transporter(s), the approach has not been explored due to the limited advancements in specific targeting of GLUTs.

**Figure 1:**
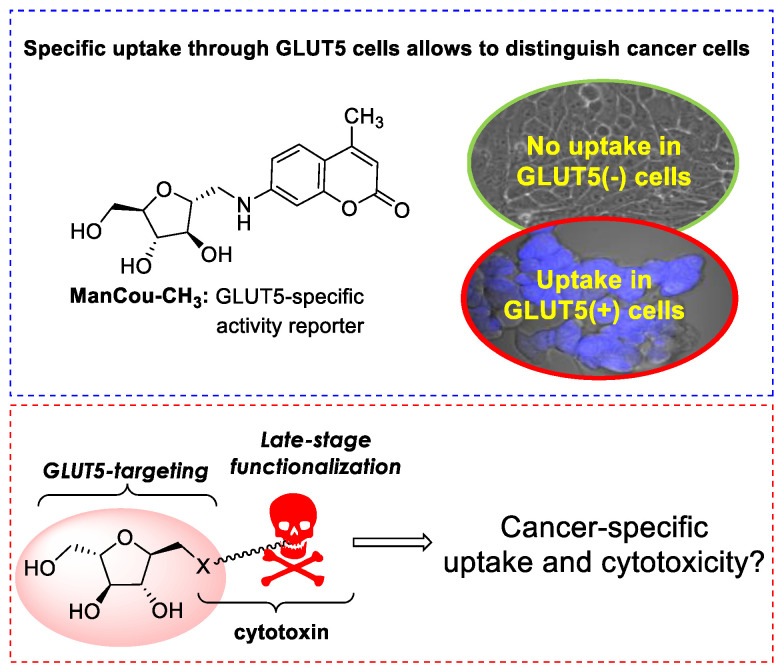
GLUT5-targeting delivery platforms amenable for late-stage functionalization with bioactive moiety for specific delivery of cytotoxic agents to GLUT5-positive cancer cells.

Prior studies in our laboratory have yielded fluorescently labeled glycoconjugates that showed high specificity and high affinity to GLUT5—fructose-specific transporter that gains expression in various cancers while tightly regulated in healthy tissues [21]. GLUT5-targeting conjugates were constructed from the 2,5-anhydro-D-mannitol (Man) as the locked fructofuranose mimic and various coumarins (Cou) as fluorescent moieties. The resulting ManCou probes (ManCou-CH_3_ representative in Figure 1) were found useful in specifically targeting GLUT5 for imaging applications and transport activity evaluation in vitro in the complete cell culture media, enabling live cell analysis [22,23]. Following this attainment, we contemplated assessing the tolerance of fructose transporter GLUT5 towards passing a bioactive moiety in a glycoconjugate form and the feasibility of achieving cancer-specific cytotoxicity. In this manuscript, we summarize our pilot studies on exploring the feasibility of transporting a bioactive agent through GLUT5 and achieving cancer-specific cytotoxicity due to the differences in the presence of GLUT5 in cancer vs. normal cells.

## 2. Results

### 2.1. Synthesis of CLB Conjugates

**Figure 2:**
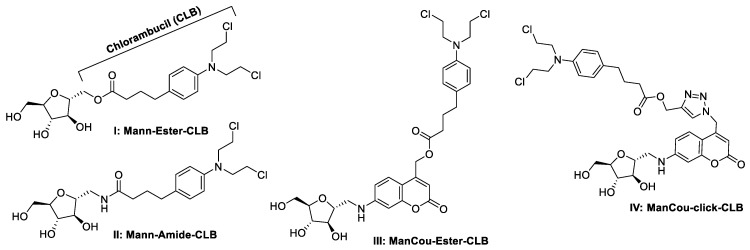
Structures of ManCou-CH_3_, chlorambucil, mannitol, mannitolamine, and chloambucil glycoconjugates.

For this proof-of-concept study, we have used chlorambucil as a bioactive target [24]. Several linkages for conjugating 2,5-anhydro-D-mannitol and CLB were assessed (Figure 2). Those included an amide and ester linkages directly to 2,5-anhydro-D-mannitol as well as analogous linkages to a ManCou conjugate that have been showing higher affinity to GLUT5 than 2,5-anhydro-D-mannitol. The two types of linkages were explored due to the feasibility of such transformation to be used as the latest stage functionalization in the drug conjugate synthesis. In addition, the “click” reaction was also explored for late-stage insertion of the bioactive agent.

**Scheme 1:**
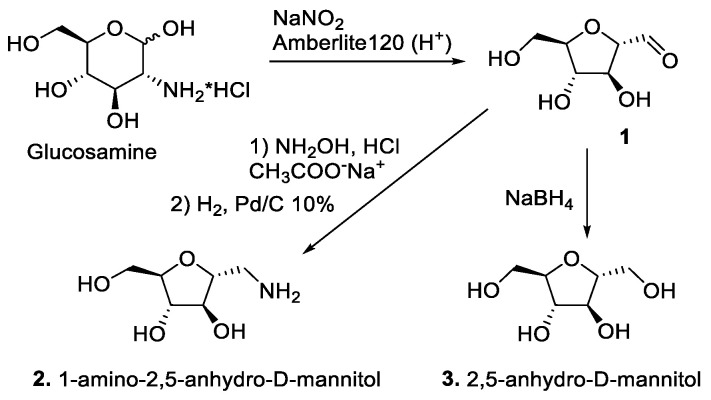
Synthesis of 2,5-anhydro-D-mannitol and 1-amino-2,5-anhydro-D-mannitol.

Scheme 1 describes the synthesis of two starting sugars to target GLUT5. 2,5-Anhydro-D-mannitol (mannitol) and 1-amino-2,5-anhydro-D-mannitol (mannitol amine) were both synthesized from D-(+)-glucosamine according to reported synthetic procedures [22]. The conversion to mannitol amine went over three steps with ~45% overall yield. The conversion to mannitol went over two steps with ~72% overall yield. All compounds were purified by silica gel column chromatography, and their structures were confirmed through NMR analysis. MS data are provided for all new compounds. High resolution MS (HRMS) is provided for final conjugates **I**–**IV**. 

**Scheme 2:**
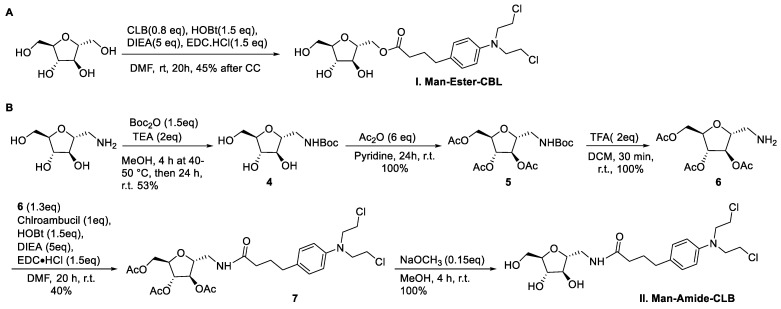
Synthesis of CLB and 2,5-anhydro-D-mannitol conjugates **I** and **II** via ester (**A**) and amide (**B**) linkages.

To obtain conjugates **I** and **II**, mannitol and mannitol amine were used in conjugation reaction with *N*,*N*-diisopropylethylamine (DIEA), respectively. While mannitol was used directly to obtain a Man–Ester–CLB conjugate **I** (Scheme 2), protection of hydroxyl group of mannitol amine was necessary to avoid off-site conjugation. We employed the strategy of orthogonal protection to mask hydroxyl groups and continue using the amine functionality for conjugation. Considering a higher reactivity of NH_2_ over OH, the amino group was protected (**4**) in the form of a carbamate (Boc). After acetylation of all hydroxyls (**5**), deprotection of the Boc-group revealed a reactive amine (**6**) that was further conjugated to CLB using DIEA to produce compound **7** [25]. The final one-step Zemplen deacetylation [25] of **7** produced the desired amide conjugate **II**.

Conjugates **I** and **II** were designed to assess the cargo-carrying capacity of mannitol as GLUT5-specific ligand. As a next step, we explored the conjugates that could provide higher affinity compounds—compounds **III** and **IV**. Here, we based the design on using ManCou as a carrier, considering a 150-fold higher affinity of these conjugates to GLUT5 due to the presence of an aromatic moiety [22]. To explore such conjugation, two strategies were used: (i) to directly conjugate ManCou with CLB through ester linkage and (ii) to explore click conjugates. Both strategies can introduce the bioactive moiety at the last step of the chemical synthesis, allowing it potentially to be used for the conjugation of chemically diverse bioactive compounds.

**Scheme 3:**
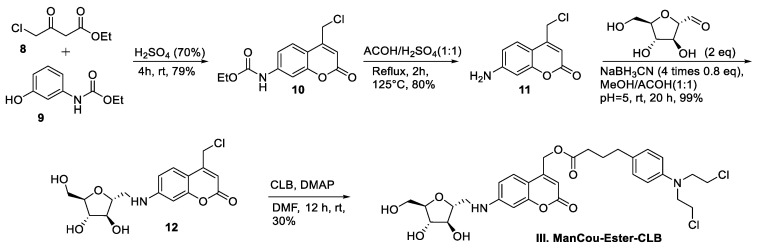
Synthesis of ManCou–Ester–CLB conjugate (**III**).

To obtain conjugate **III**, we explored the formation of ester linkage through a direct displacement of a leaving group at the exocyclic methylene of coumarin. For this part, the 7-amino-4-chloromethylcoumarin was synthesized according to the reported procedure using ethyl 4-chloro-3-oxobutanoate (**8**) and N-protected 3-aminophenol (**9**) [26]. Cyclization of **8** and **9** in acidic conditions, followed by acid-mediated cleavage of N-ethyl formate from then formed compound (**10**) has provided the desired 7-amino-4-chloromethylcoumarin (**11**) in high yield (Scheme 3). The following reductive amination resulted in the corresponding mannitol conjugate (**12**) that was used for conjugation with CLB in basic conditions. After column and HPLC purification, the resulting ManCou–Ester–CLB conjugate **III** was obtained in 20% yield.

**Scheme 4:**
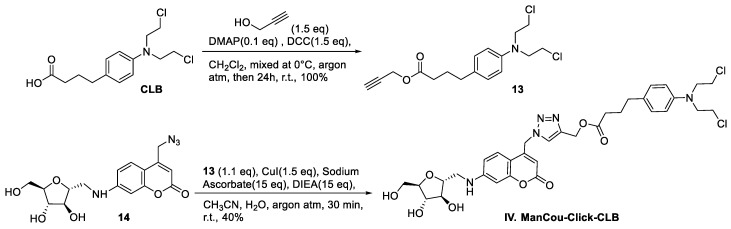
Synthesis of a click conjugate (**IV**).

We further explored a synthesis of conjugate **IV**. The orthogonal reactivity between azide and alkyne groups has been demonstrated as a practical synthetic tool for the modification of carbohydrates, waiving the requirement for the protection of free hydroxyl groups [27]. So, we proceeded with forming two components for the “click” reaction: ManCou-azide (**14**) and alkyne ester of CLB (**13**) (Scheme 4). Compound **14** was obtained through the direct displacement of chloride of **12**. Alkyne ester of CLB was obtained through standard DCC-mediated coupling of CLB and propargyl alcohol. For the construction of the final conjugate **IV** through click reaction, we adapted a literature method employing an excess of CuI, sodium ascorbate, and DIEA under aqueous conditions [28]. The conjugate **IV** was obtained in moderate yield on the scale sufficient for further biological evaluations.

Overall, both synthetic strategies—direct displacement and click reaction—were proven to be effective in introducing the bioactive moiety at the last step of the synthesis. While limited to conjugates of CLB, these strategies can prove effective in the formation of conjugates of more chemically labile biologically active agents.

### 2.2. Assessing GLUT5 Levels in Cells

Considering a strong link between GLUT5 activity and breast cancer [19], we have selected breast cancer cells for biochemical evaluation of probes **I**–**IV**: GLUT5-positive breast adenocarcinoma MCF7 cell line, GLUT5-positive human breast invasive ductal carcinoma MDA-MB-231 cell line, and GLUT5-negative normal breast 184B5 cells.

**Figure 3:**
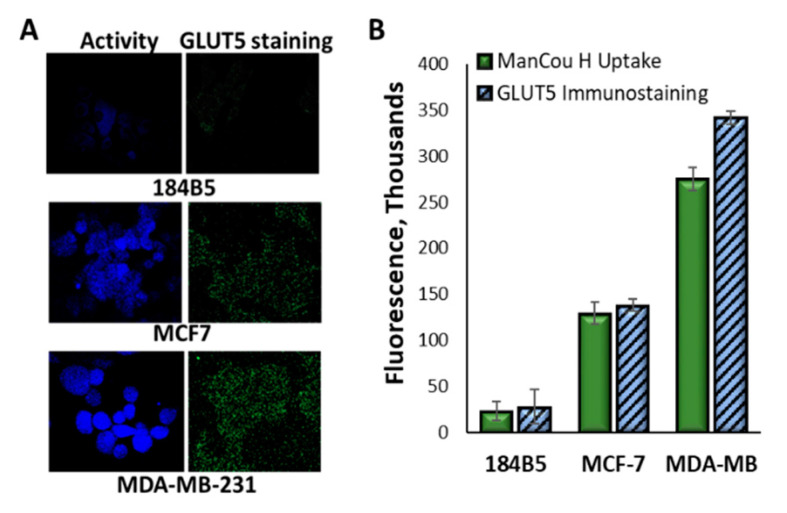
Comparative analysis of ManCou uptake and membrane GLUT5 levels in MCF7, MDA-MB-231 and 184B5 cells. (**A**) Confocal microscope images of cells treated with ManCou-H (blue fluorescence) and GLUT5-specific antibody (green fluorescence). Images obtained with 60X objective. ManCou-H imaged using DAPI filter (ex/em: 405/465 nm), GLUT5 antibody imaged using Alexa488 filter (ex/em: 488/565 nm). Images were recorded at the same laser intensity and exposure time. (**B**) Corrected total cell fluorescence (CTCF) derived from fluorescence images after quantification with ImageJ. CTCF = integrated density − (Area of selected cell X mean fluorescence of background readings).

We used specific labeling of GLUT5 in the cellular membrane with the corresponding antibody and GLUT5-specific ManCou-CH_3_ probe to assess the relative membrane levels and activity of GLUT5, respectively. After immunostaining, fluorescence was recorded using a confocal microscope and quantified using ImageJ. CTCF values were derived for each cell (Figure 3A,B). Overall, the analysis of GLUT5 expression in the membrane reflected a significant difference in GLUT5 levels between three cell lines, with 184B5 cells showing the minimal presence of the transporter in the membrane. Relative to 184B5, expression of GLUT5 in the membrane was measured ~5-fold higher for MCF7 cells and 17-fold higher for MDA-MB-231 cells.

We measured GLUT5 transport activity by monitoring the accumulation of fluorescence ManCou-H probe in cells. After short 10 min incubation of live cells with 25 µM ManCou-H probe in complete culture medium, fluorescence was recorded using a confocal microscope and quantified using ImageJ. The resulting difference in the ManCou-H uptake reflected the relative activity of GLUT5 between cells. The uptake correlated well with the differences in membrane GLUT5 levels, with the exception of the MDA-MB-231 cells, where a higher accumulation of the probe (more active uptake) was observed. Overall, the three cell lines can be categorized with respect to the expected GLUT5-assisted accumulation of our bioactive conjugates, with the negligible uptake expected for normal 184B5 cells and the highest uptake expected for malignant MDA-MB-231 cells.

### 2.3. Conjugates I–IV Show GLUT5-Dependent Uptake during Short Incubations

**Figure 4:**
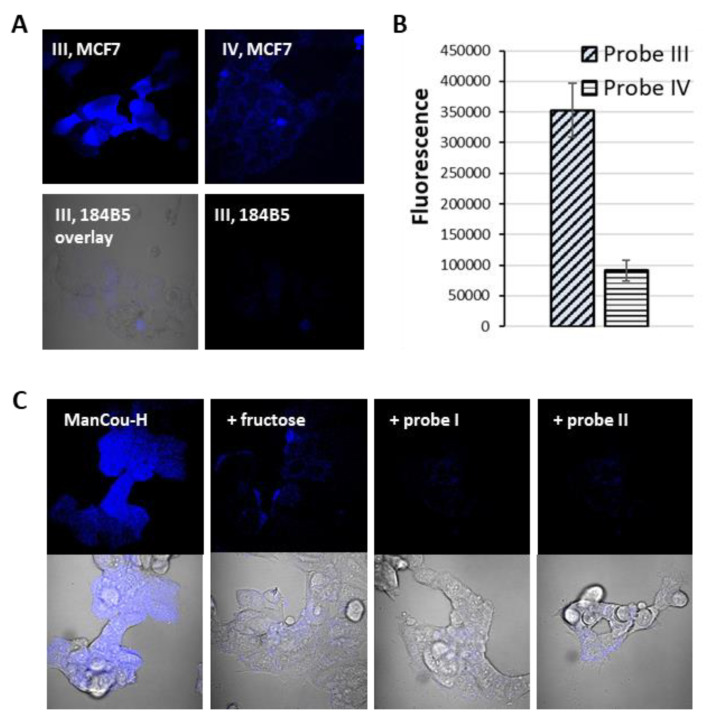
Uptake analysis for fluorescent conjugates **III** and **IV**. (**A**) Comparative uptake in MCF7 vs 184B5 cells shows the involvement of GLUT5 in the uptake of **III** and **IV**. (**B**) Comparative uptake efficiency of **III** and **IV** in MCF7 cells. Corrected total cell fluorescence (CTCF) derived from fluorescence images after quantification with ImageJ. CTCF = integrated density − (Area of selected cell X mean fluorescence of background readings). (**C**) Competitive inhibition of ManCou-H (5 µM) uptake by fructose, probe **I**, and probe **II** (500 µM): fluorescence and bright-field/fluorescence overlay images. All fluorescence and bright fields images were obtained using confocal microscope, with 60X objective using. Florescence images were obtained with DAPI filter (ex/em: 405/465 nm). Images were recorded at the same laser intensity and exposure time.

We have further assessed whether conjugates **I**–**IV** can be taken up through GLUT5. Fluorescent conjugates **III**–**IV** were directly incubated with GLUT5-positive MCF7 cells, and GLUT5-negative 184B5 cells. Probe uptake was evaluated through analysis of fluorescent images acquired with a confocal microscope. After incubating cells with 25 µM solutions of probes, we found both conjugates to induce significant fluorescence in MCF7 cells but not 184B5 cells (Figure 4A). The uptake of two probes appears to differ in efficiency, with ester conjugate **III** inducing >3-fold higher fluorescence than **IV**. Considering that both conjugates encompass the same fluorophore, a lesser GLUT5-uptake efficiency appears to be evident for conjugate **IV**. With respect to 184B5 cells, we have detected residual fluorescence with conjugate **III** and no fluorescence for conjugate **IV** (image not included). As neither conjugate was taken up by 184B5 cells, the uptake can be attributed to GLUT5 activity in MCF7 cells.

In order to assess the ability of conjugate **I** and **II** to pass into the cell through GLUT5, a competitive uptake with ManCou-H probe was carried out. MCF7 cells were incubated in parallel with ManCou-H, ManCou-H + probe **I**, and ManCou-H + probe **II**. 100-fold higher concentration of probe **I** or probe **II** was used to compensate for the ~150-fold higher binding of ManCou-H over mannitol to GLUT5 [22]. The analogous inhibition with fructose has been carried out as a control experiment. After co-incubating 5 µM ManCou-H with 500 µM fructose, probe **I** or probe **II**, we have observed a significant decrease in ManCou-H fluorescence, suggesting the direct competition between substrates and ManCou-H for GLUT5-mediated uptake.

### 2.4. CLB Conjugates Show Structure-Activity Relationship

With a better understanding of the difference in GLUT5 expression and activity in selected cell lines, we moved forward with assessing whether the undertaken modification to deliver a drug to GLUT5 can improve the activity and specificity of CLB. We carried out a continuous exposure to conjugates **I**–**IV** and CLB as control (the dose of the bioactive compounds was given at time zero and measured cytotoxicity after 24 h and 48 h using a 96-well plate MTS cell proliferation assay. Concentrations within 0.1–500 µM were selected for the analysis. All five compounds were evaluated in one 96-well plate. Independent experiments were carried out three times. Each plate contained a drug-free row that was used for deriving a relative cell growth inhibition.

As summarized in Table 1 and Figure 4 (24 h data presented), all conjugates appear to induce cytotoxicity equivalent to that of CLB in MCF7 and MDA-MB-231 cells after 24 h or 48 h incubation. Subtle differences can be observed between cytotoxic responses of two cancer cell lines. Thus, more aggressive MDA-MB-231 cells appear to respond better to ester conjugates **I** and **III** than to CLB, **II**, or **IV**. While this can relate to the higher presence and activity of GLUT5 in MDA-MB-231 cells, the overall differences in cytotoxicity are not as significant as the differences in GLUT5 levels between cell lines.

**Figure 5:**
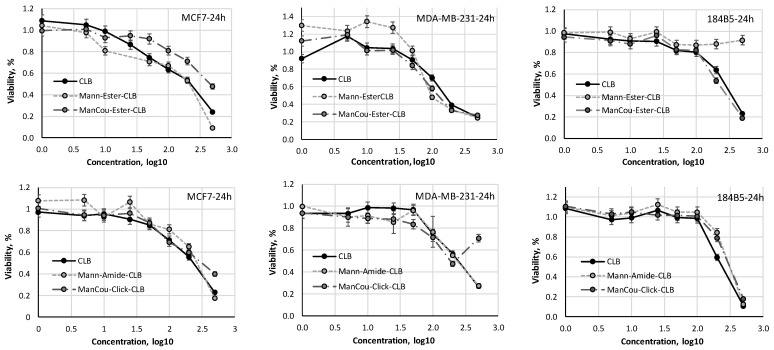
Cytotoxicity data for CLB and conjugates **I**–**IV** (1–500 µM) in MCF7 (breast adenocarcinoma), MDA-MB-231 (breast invasive ductal carcinoma) and 184B5 (normal breast) cells after 24 h. Cytotoxicity data measured with MTS assay using 1–500 µM CLB or probe concentrations. Untreated cells were used as a control to derive a relative % viability. Concentrations are presented as log10 (1 µM treatment concentration is presented as zero point on x-axis). Error bars derived from independent *n* = 3 repetitions of every experiment.

While moderately impacting the activity of CLB, sugar conjugation has contributed to the selectivity of one of the test conjugates—Man–Ester–CLB (**I**). Thus, while significant cytotoxicity was observed for CLB and all probes in MCF7 and MDA-MB-231 cells, normal breast 184B5 cells were not impacted by Mann–Ester–CLB (**I**) conjugate. Cytotoxicity of other probes (**II**–**IV**) in the 184B5 cell line was similar to that of CLB (Figure 5). Cytotoxicity data for CLB and conjugates **I**–**IV** (1–500 µM) in MCF7 (breast adenocarcinoma), MDA-MB-231 (breast invasive ductal carcinoma), and 184B5 (normal breast) cells after 24 h. Cytotoxicity data were measured with MTS assay using 1–500 µM CLB or probe concentrations. Concentrations are presented as log10. Error bars derived from independent *n* = 3 repetitions of every experiment.

### 2.5. Analysis of CLB Conjugate Hydrophobicity

**Figure 6:**
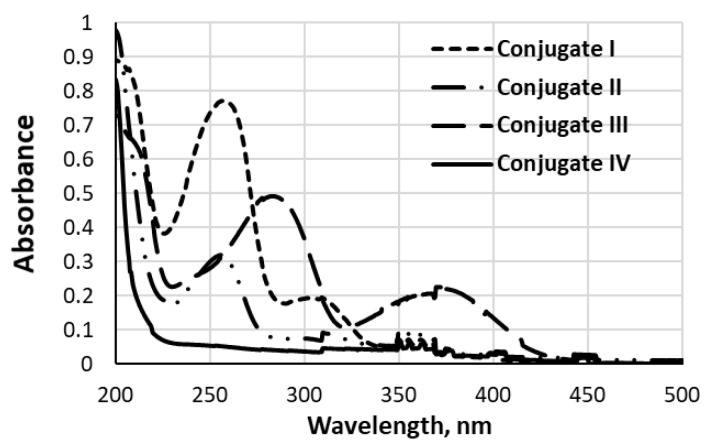
UV absorbance of aqueous phase from octanol/water extraction.

Hydrophobicity of conjugates **I**–**IV** was evaluated by assessing their relative water solubility by *n*-octanol/water partition according to the reported protocol [29]. Figure 6 depicts UV spectra obtained for aqueous parts after partition with octanol. As can be seen, there is a significant decrease in the substrate concentration in aqueous (PBS) extract upon an increase of the compound molecular mass/carbon content. Namely, while high presence is detected for the ester conjugate **I**, compound **IV** was completely extracted by the octanol. Hence, conjugates can be arranged in the order of relative hydrophilicity as follows: **I** > **II** > **III** > **IV**.

## 3. Discussion

While GLUTs have been viewed as important therapeutic targets for several decades, as yet the progress of specific targeting of GLUTs for drug delivery is minimal [16]. Two factors that contribute to this limitation are the lack of approaches to target only disease-relevant transport(s) and a little understanding of transport capacity. We report here the first study that explores the feasibility of delivering a bioactive cargo through one cancer-relevant transporter explicitly. We have selected fructose transporter GLUT5 as a target due to its direct relevance to cancer, tightly regulated expression in normal cells, and our capability to specifically deliver small fluorescent molecular probes through this transporter into the cell [22].

Using GLUT5-directing 2,5-anhydro-D-mannitol (mannitol), we have aimed to explore the feasibility of delivering a bioactive cargo through GLUT5 and achieving cancer specificity of the cytotoxic response. Chlorambucil (CLB) is DNA-alkylation-inducing nitrogen mustard used under the trade name Leukeran approved as an effective antineoplastic agent in clinical treatment against manifold malignant and nonmalignant diseases, namely chronic lymphatic leukemia, lymphomas, and advanced ovarian and breast carcinomas [24]. Application of CLB has been restricted due to its toxicity arising from the lack of specificity to tumors. Using chlorambucil (CLB) as a bioactive cargo, we synthesized direct ester and amide conjugates of 2,5-anhydro-D-mannitol (**I** and **II**, Figure 1). To enhance the affinity of conjugate-GLUT5 interaction, we have introduced an aromatic spacer (coumarin) that has been previously shown to contribute up to 100-fold to the probe-GLUT5 interaction [22]. The synthetic approaches considered were based on achieving late-stage functionalization with bioactive moiety and included esterification and “click” conjugation, resulting in conjugates **III** and **IV** (Figure 2).

For biological studies, we have selected three cell lines with differential expression and activity of GLUT5. MCF7 and MDA-MB-231 cells are breast cancer cell lines showing high levels of GLUT5 activity [30]. Using immunofluorescence, we have established relative levels of GLUT5 expression within the membrane for these two cell lines, reporting >3-fold higher levels in more aggressive MBA-MB-231 cells. Using our GLUT5-specific fluorescent ManCou-H probe, we have established that the activity of GLUT5 parallels the membrane expression levels, suggesting that the highest uptake of GLUT5-delivered conjugate would be expected for MDA-MB-231 cells, followed by MCF7 cells (Figure 3). With only basal levels of GLUT5 detected in normal breast 184B5 cells, the measured membrane expression of GLUT5 in MCF7 and MDA-MB-231 cells was, respectively, 5- and 17-fold higher. This set of cell lines provided a good platform to assess the impact of directing CLB to GLUT5.

We have further assessed whether the synthesized conjugates can be taken up by the cell through GLUT5. As not all conjugates exhibit fluorescence, two different approaches were used for the analysis. Fluorescent conjugate **III** and **IV** were directly evaluated for the uptake in GLUT5-positive MCF7 cells and GLUT5-negative 184B5 cells (Figure 4A,B). After 10 min incubation of cells with 20 µM **III** or **IV**, significant levels of probe-induced fluorescence were observed using a confocal microscope. The fluorescence levels were over three-fold higher for probe **III** than for **IV**. The later could be contributed by the slower uptake kinetics, as was observed with ManCou probes bearing hydrophobic moieties at the C4 of a coumarin [22]. In contrast to GLUT5-positive MCF7 cells, there was no accumulation of fluorescence in GLUT5-negative 184B5 cells. The latter provided direct evidence for the direct involvement of GLUT5 in the uptake of **III** and **IV** over a short incubation time.

The involvement of GLUT5 in the uptake of non-fluorescent **I** and **II** was assessed by measuring the ability of these probes to competitively inhibit the uptake of ManCou-H as a GLUT5-specific probe. We found that co-incubation of ManCou-H with probes **I** and **II** induced almost compete inhibition of ManCou-H uptake (Figure 4C). The impact was analogous to that of fructose, suggesting that all tested substrates compete for GLUT5.

After validating the ability of **I**–**IV** to pass through GLUT5, their cytotoxicity was assessed using an MTS assay. Measuring the level of cell death over a range of concentrations reflected sufficient changes to derive the IC_50_ values for every conjugate (Figure 5, Table 1). We have observed that cytotoxic response was both time-dependent and cell type-dependent. Thus, prolonged treatment of cells (48 h) induced a stronger response in cancer cells but did not impact normal cells. Interestingly, we did not observe any correlation between the relative differences in GLUT5 activity and cytotoxic response for MCF7 and MDA-MB-231 cells. We did, however, observe a remarkable selectivity for conjugate **I**. Thus, while being very active in cancer cells, this conjugate showed no cytotoxicity in 184B5 cells after 24 h or 48 h treatment. Conjugates **II**–**IV** were observed to be cytotoxic for normal cells, albeit with relatively higher IC_50_ values.

With the same targeting moiety employed in all four test conjugates, the observed differences in cytotoxicity and selectivity of these conjugates directly highlight the structure-activity relationship. Stringent requirements to substrate properties have been previously observed for GLUTs. Earlier observations include the loss for GLUT-mediated uptake upon functionalization of glucose or fructose hydroxyls [5], highlighting the key role of specific H-bonding interactions of GLUT-substrate recognition. Lately, more evidence on requirements to cargo has emerged. Namely, while GLUTs-dependent uptake is evident for fluorescent coumarin conjugates of glucose or fructose, the lack of uptake was observed for coumarin conjugates bearing carboxylate moieties, suggesting discrimination against charged species by GLUTs [22]. Likewise, fluorescein conjugate of mannitol did not show GLUT-dependent uptake [30].

Considering the fundamental role of GLUTs as transporters of hydrophilic substrates through a hydrophobic cellular membrane, it is highly feasible that cytotoxicity of conjugates **II**–**IV** in normal 184B5 cells results from the lack of GLUT5-mediated uptake. We have surmised that the loss in GLUT5-mediated uptake may be driven by enhanced hydrophobicity of conjugates **II**–**IV** as compare to the conjugate **I**. Indeed, measuring the levels of conjugates in water after octanol/water extraction clearly reflected the lower concentration of **II**–**IV** in an aqueous phase compared to conjugate **I**. The hydrophobicity of substrates increased in the order **I** < **II** < **III** < **IV**, posing the conjugate **I** as most hydrophilic and conjugate **IV** as least hydrophilic. Respectively, the loss of GLUT5-involvement in the internalization of hydrophobic conjugates and the shift towards passive diffusion through the cellular membrane with increased hydrophobicity would be expected to diminish selectivity between normal and cancer cells.

## 4. Materials and Methods

### 4.1. General Methods

All reagents were used as received unless otherwise stated from Sigma-Aldrich (St Lois, MO, USA), TCI America (New Jersey, NJ, USA), Alfa Aesar (Haverhill, MA, USA), Ark Pharm (Arlington Heights, IL, USA), or Chem-Impex International (Wood Dale IL, USA). Analytical TLC was carried out on commercial SiliCycle SiliaPlate^®^ 0.2 mm F254 plates. Preparative silica chromatography was performed using SiliCycle SiliaFlash^®^ F60 40–63 μm (230–400 mesh). Final purification of compounds was achieved with Agilent-1200 HPLC (high-pressure liquid chromatography) using reverse-phase semi-preparative column (Phenomenex^®^ Luna^®^ 10 µm C18(2) 100 Å, LC Column 100 × 10 mm, Ea). ^1^H, ^13^C, and ^19^F NMR spectra were recorded at room temperature with a Varian Unity Inova 400 MHz spectrometer. CDCl_3_, CD_3_OD, and DMSO-d_6_ were used as solvents and referenced to the corresponding residual solvent peaks (7.26 and 77.16 ppm for CDCl_3_, respectively; 3.31 and 49.0 ppm for CD_3_OD, respectively; 2.50 and 39.52 ppm for DMSO-d_6_, respectively) [31]. The following abbreviations are used to indicate the multiplicity: s—singlet; d—doublet; t—triplet; q—quartet; m—multiplet; b—broad signal; app—approximate. The coupling constants are expressed in Hertz (Hz). The multiplicity of carbon atoms was determined by DEPT-135 experiment. The high-resolution (HR) MS data (ESI) were obtained using a Thermo Fisher Orbitrap Elite™ Hybrid Ion Trap-Orbitrap Mass Spectrometer at Chemical Advanced Resolution Methods (ChARM) Laboratory at Michigan Technological University. UV-vis spectra were recorded on a Cary 100 Bio-spectrophotometer from Agilent Technologies. Fluorescence imaging was done with EVOS FLAuto inverted microscope. Confocal images were taken with Olympus FluoViewTM FV1000 using the FluoView software. RPMI-1640, Penicillin/Streptomycin, FBS (Fetal Bovine Serum), 25% Trypsin-EDTA (1×), and PBS (phosphate buffered saline solution) were purchased from Life Technologies (Carlsbad, CA, USA). MEM Non-Essential Amino Acids 100 × were purchased from Quality Biological (Gaithersburg, MD, USA). Sterile DMSO (25-950-CQC, 250 mL) was purchased from Sigma-Aldrich (St Lois, MO, USA). MCF7, MDA-MB231, 184B5 cells were purchased from ATCC (Manassas, VA, USA). MTS assay was performed using CellTiter 96^®^ AQueous One Solution Cell Proliferation Assay from Promega (Madison, WI, USA). GLUT5 primary Mouse Monoclonal antibody (sc-271055) was purchased from Santa Cruz Biotechnology Inc. (Dallas, TX, USA). The secondary antibody Goat anti-Mouse Alexa Fluor Plus 488 (A327230 was purchased from ThermoFisher Scientific (Waltham, MA, USA).

### 4.2. Organic Synthesis

2,5-Anhydro-2-carbaldehyde-D-mannitol (**1**) [32]: D-Glucosamine hydrochloride (4.00 g, 18.5 mmol) was dissolved in water (100 mL) and stirred at room temperature for 5 h. Sodium nitrite (3.19 g, 45.3 mmol) was then added, followed by cautious addition of Amberlite 120 H^+^ resin (90 g) by portion. The reaction mixture was maintained on ice bath for 4 h. The completion of the reaction was confirmed by TLC. After the reaction, the resin was removed by filtration, and the solution was then neutralized by sodium carbonate. The remaining solution was vacuum dried, and methanol was added to the residue to precipitate the inorganic salts. After removing the salts by filtration, the solution was vacuum dried to get the product as a yellow sticky solid (2.49 g, 70%) that was used directly without further purification.

((2*R*,3*S*,4*S*,5*R*)-3,4-Dihydroxy-5-(hydroxymethyl)tetrahydrofuran-2-yl)methyl 4-(4-(bis(2- chloroethyl)amino) phenyl)butanoate (**I**, Man–Ester–CLB) [25]. To a solution of (2*R*,3*S*,4*S*,5*R*)-2,5-bis(hydroxymethyl)tetrahydrofuran-3,4-diol(2,5-anhydro-2-carbaldehyde-D-mannitol) (177 mg, 1.07 mmol) in DMF (5 mL), chlorambucil (252 mg, 0.828 mmol), HOBt (210 mg, 1.60 mmol), DIEA (0.896 mL, 5.35 mmol), and EDC•HCl (300 mg, 1.60 mmol) were added and stirred at room temperature for 22 h. The mixture was partitioned between EtOAc and water (50:50 mL). Organic layer was separated, and aqueous phase was extracted with EtOAc (2 × 20 mL). Organic phases were combined, washed with brine, dried over Na_2_SO_4_, and concentrated under vacuum. The resulting residue was purified by silica gel chromatography, using Methanol/DCM (1:4), to yield the target compound **I** (163 mg, 45%) as a syrup: ^1^H NMR (400 MHz, CD_3_OD) δ 7.46 (t, *J* = 8 Hz, 2H), 7.11 (t, *J* = 8 Hz, 2H), 4.99 (m, 1H), 4.51 (m, 2H), 3.94 (m, 1H), 3.21–3.62 (m, 8H), 2.74 (t, *J* = 4 Hz, 2H), 2.57 (t, *J* = 4 Hz, 2H), 2.32–2.40 (m, 2H). HRMS (M + H^+^): calc’d 389.1686, obs’d 389.1679

*Tert*-butyl(((2*R*,3*S*,4*S*,5*R*)-3,4-dihydroxy-5-(hydroxymethyl)tetrahydrofuran-2-yl)methyl)carbamate (**4**). To a solution of mannitolamine [22] (480 mg, 3 mmol) in MeOH (10 mL), TEA (0.3 mL, 6 mmol) and Di-*tert*-butyl dicarbonate (0.96 g, 4.5 mmol) were added. Reaction was maintained at 47 °C for 4 h and at room temperature overnight. After completion, reaction mixture was concentrated under vacuum and the resulting residue was purified by silica gel chromatography, using Methanol/DCM (1:9) as eluent, to yield **4** (390 mg, 50%) as a white solid: ^1^H NMR (400 MHz, D_2_O) δ 8.31 (s, 1H), 3.8–3.9 (m, 2H), 3.76 (m, 1H), 3.5–3.65 (m, 2H), 3.04–3.21 (dd, 1H), 1.30 (s, 9H). MS (M + H^+^, ESI): 264.1

(2*R*,3*R*,4*R*,5*R*)-2-(Acetoxymethyl)-5-(((*tert*-butoxycarbonyl)amino)methyl)tetrahydrofuran-3,4-diyl triacetate (**5**) [25]: To a solution of **4** (1.745 g, 6.635 mmol) in pyridine (30 mL), acetic anhydride (3.80 mL, 40 mmol) was added. The mixture was stirred at room temperature for 24 h. TLC in EtOAc/Hex (1:4) using vanillin and ninhydrin staining confirmed the completion of the protection reaction. Pyridine was removed by concentration under vacuum. The resulting syrup was extracted by EtOAc and water (2 × 15 mL). The organic extract was washed with brine (1 × 15 mL), dried by Na_2_SO_4_ and concentrated under vacuum to yield compound **5** as a dark yellow solid: ^1^H NMR (400 MHz, CDCl_3_), δ 8.50 (s, 1H), 5.02–5.10 (m, 3H), 4.96 (m, 1H), 4.00–4.21 (dd, 2H), 3.25–3.50 (dd, 2H), 2.08 (s, 3H), 2.06 (s. 6H), 1.44 (s, 9H). HRMS (M + H^+^): calc’d 389.1686, obs’d 389.1679.

(2R,3R,4R,5R)-2-(Acetoxymethyl)-5-(aminomethyl)tetrahydrofuran-3,4-diyldiacetate (**6**). To a solution of **5** (0.294 g, 0.75 mmol) in DCM, TFA (0.172 g, 1.5 mmol) was added. The mixture was stirred at room temperature for 30 min. After reaction completion (monitored by TLC in EtOAc/Hex (1:4) using vanillin and ninhydrin staining) solvent was concentrated to dryness and the resulting brown residue was used without purification.

(2*R*,3*R*,4*R*,5*R*)-2-(Acetoxymethyl)-5-((4-(4(bis(2-chloroethyl)amino)phenyl)butanamido)methyl) tetrahydrofuran-3,4-diyl diacetate **(7) [25]**: To a solution of **6** (700 mg, 2.42 mmol) in DMF (5 mL), chlorambucil (566 mg, 1.86 mmol), HOBt (0.337 g, 2.79 mmol), DIEA (1.20 g, 9.31 mmol), and EDC•HCl (1.20 g, 2.79 mmol) were added and stirred at room temperature for 22 h. The mixture was partitioned between EtOAc and water (50:50 mL). Organic layer was separated, and aqueous phase was extracted with EtOAc (2 × 20 mL). Organic phases were combined, washed with brine, dried over Na_2_SO_4_, and concentrated under vacuum. The resulting residue was purified by silica gel chromatography, using Hexane/Ethyl acetate (7:3) as eluent, to yield **7** (129 mg, 40%) as a syrup: ^1^H NMR (400 MHz, CD_3_OD) δ 8.00 (s, 1H), 6.60 (d, *J* = 8 Hz, 2H), 7.05 (d, *J* = 8 Hz, 2H), 5.75 (m, 1H), 5.00–5.20 (m, 2H), 4.07–4.22 (m, 2H), 3.60–3.68 (m, 2H), 2.86–2.94 (dd, 8H), 2.18 (t, *J* = 4 Hz, 2H), 2.54 (t, *J* = 4 Hz, 2H), 2.08 (s, 3H), 2.02 (s, 6H), 1.24 (p, 2H). MS (M + H^+^, ESI): 575.1.

4-(4-(bis(2-chloroethyl)amino)phenyl)-*N*-(((2*R*,3*S*,4*S*,5*R*)-3,4-dihydroxy-5-(hydroxymethyl)tetrahydrofuran-2-yl)methyl)butanamide (**II**, Man–Amide–CLB). To the solution of **7** (0.273 mg, 0.474 mmol) in methanol, sodium methoxide (3 mg, 0.074 mmol) was added and stirred at room temperature for 4 h. The completion of deprotection was verified by TLC analysis in hexane/ethyl acetate (7:3) as eluent and yielded compound **II** as a yellowish solid (0.250 g, 99%). ^1^H NMR (400 MHz, CD_3_OD) δ 8.00 (s, 1H), 6.60 (d, *J* = 8 Hz), 7.05 (d, *J* = 8 Hz, 2H), 5.75 (m, 1H), 5.00–5.20 (m, 2H), 4.07–4.22 (m, 2H), 3.60–3.68 (m, 2H), 2.86–2.94 (dd, 8H), 2.18 (t, *J* = 4 Hz, 2H), 2.54 (t, *J* = 4 Hz, 2H), 1.24 (p, 2H). HRMS (M + H^+^): calc’d 389.1686, obs’d 389.1679

(3-Hydroxyphenyl)carbamic acid ethyl ester (**9**) was previously synthesized in [33]: 3-Aminophenol (10.0 g, 91.6 mmol) and ethyl acetate (40 mL) were refluxed for 1 h with vigorous stirring. Ethyl chloroformate (4.4 mL, 45.8 mmol) was then added via addition funnel over a 30 min. The reaction mixture was refluxed for an additional hour and then allowed to cool to room temperature. Upon cooling, a grey/white precipitate formed within the flask. The precipitate was removed via filtration and washed with ethyl acetate (3 × 100 mL). The combined filtrates were concentrated to afford the target compound as a grey solid (9.50 g, 58%) that was used without further purification. ^1^H NMR (400 MHz, DMSO-d6): *δ*, 9.45 (s, 1H), 9.29 (bs, 1H), 7.04–7.00 (m, 2H), 6.86–6.84 (m, 1H), 6.39–6.36 (ddd, *J1* = 1.2, *J2* = 2.4, *J3* = 8.0, 1H), 4.12–4.07 (q, *J* = 7.2, 2H), 1.24–1.21 (t, *J* = 7.2, 3H).

Ethyl (4-(chloromethyl)-2-oxo-2H-chromen-7-yl)carbamate (**10**) was previously synthesized in [34]: (3-Hydroxyphenyl) carbamic acid ethyl ester (**9**) (2.00 g, 11 mmol) and ethyl 4-chloro-3-oxobutanoate (2.17 g, 13.20 mmol) were added to a 100 mL round-bottom flask equipped with a stir bar. 70% H_2_SO_4_ (60 mL) was added, and the mixture was stirred at room temperature for 4 h. The reaction mixture was then poured over 100 mL of crushed ice to give a bright yellow precipitate. The solid was filtered and washed several times with distilled water to remove acid. It was left for several hours to dry and then yellowish white precipitate was collected (2.48 g, 80%). ^1^H NMR (400 MHz, DMSO-d6): *δ* 10.14 (s, 1H), 7.73–7.71 (d, *J* = 2.4, 1H), 7.56 (m, 1H), 7.36–7.39 (m, 1H), 6.48 (s, 1H), 4.94 (s, 2H), 4.12–4.14 (q, *J* = 7.2, 2H), 1.20–1.24 (t, *J* = 7.2, 3H).

7-Amino-4-(chloromethyl)-2*H*-chromen-2-one (**11**) was previously synthesized in [34]: (2.48 g, 8.52 mmol) of compound (**10**) was added to a 50 mL round-bottom flask, followed by H_2_SO_4_ conc. (8 mL) and glacial AcOH (8 mL). The reaction mixture was heated to 125 °C for 2 h under reflux condenser, after which it was cooled to room temperature and poured over 300 mL of crushed ice. The resulting suspension was neutralized with 4M KOH, affording a yellow precipitate. The precipitate was filtered and collected to give the target compound as a fine yellow powder (1.40 g, 80%). ^1^H NMR (400 MHz, DMSO-d6): *δ* 10.14 (s, 1H), 7.43–7.45 (d, *J* = 2.4, 1H), 6.54–6.56 (m, 1H), 6.41 (s, 1H), 7.14–7.19 (m, 1H), 6.48 (s, 1H), 4.34 (s, 2H).

4-(Chloromethyl)-7-((((2*R*,3*S*,4*S*,5*R*)-3,4-dihydroxy-5-(hydroxymethyl)tetrahydrofuran-2-yl)methyl)amino)-2*H*-chromen-2-one acetic acid salt (**12**) [35]: 2,5-anhydro-2-carbaldehyde-D-mannitol (800 mg, 4.8 mmol) and compound **11** (500 mg, 2.40 mmol) were dissolved in methanol (40 mL). AcOH (4.0 mL) was added to adjust the pH to <6, and the mixture was stirred at room temperature for 10–15 min, followed by portion-wise addition of NaBH_3_CN (4 x 120 mg, 1.92 mmol, every 30–40 min). The reaction mixture was stirred at room temperature for overall 24 h. The mixture was concentrated to dryness under reduced pressure, absorbed on silica gel, and purified by column chromatography on silica gel eluting with 0%−10% methanol in CH_2_Cl_2_. The product was obtained as an acetic acid salt, yellow foam in 90% yield (840 mg): ^1^H NMR (400 MHz, CD_3_OD): δ 7.45–7.47 (d, *J* = 9.2, 1H), 6.6–6.68 (dd, *J_1_* = 2.4, *J_2_* = 8.8, 1H), 6.48 (d, *J* = 2.4, 1H), 6.13–6.15 (d, *J* = 2.4, 1H), 5.45 (s, 1H), 4.71 (m, 2H), 3.93–3.96 (m, 2H), 3.87–3.88 (m, 1H), 3.60–3.69 (m, 2H), 3.50 (s, 1H), 3.29 (m, 2H). MS (M + H^+^): 356.1

(7-((((2*R*,3*S*,4*S*,5*R*)-3,4-dihydroxy-5-(hydroxymethyl)tetrahydrofuran-2-yl)methyl)-amino)-2-oxo-2*H*-chromen-4-yl)methyl 4-(4-(bis(2-chloroethyl)amino)phenyl)butanoate (III, ManCou–Ester–CLB): to the solution of 12 (234 mg, 0.66 mmol) in dry DMF (5 mL) under argon, chlorambucil (200 mg, 0.66 mmol) and DMAP (80 mg, 0.66 mmol) was added, and the mixture was stirred at room temperature for 24 h. The mixture was concentrated and purified by column chromatography, using methanol/CH_2_Cl_2_ up to 30% to yield III as a light-yellow syrup. ^1^H NMR (400 MHz, CD_3_OD): δ 7.37–7.39 (d, *J* = 9.2, 1H), 6.95–7.05 (m, 2H), 6.60–6.70 (m, 2H), 6.55 (d, *J* = 4, 1H), 6.05 (m, 2H), 5.22 (s, 2H), 4.90 (m, 4H), 4.61 (m, 1H), 3.93–3.96 (m, 1H), 3.69 (s, 1H), 3.61–3.63 (m, 8H), 3.54–3.56 (m, 2H), 3.29–3.32 (m, 2H), 2.40–2.44 (dd, 2H), 2.50–2.54 (dd, 2H), 1.81 (m, 2H). HRMS (ESI): m/z [M + H]^+^ calc’d for C_30_H_36_Cl_2_N_2_O_8_: 622.18487; found: 622.18446.

Prop-2-yn-1-yl 4-(4-(bis(2-chloroethyl)amino)phenyl) butanoate (**13**) [36]: Prop-2-yn-1-ol (55 mg, 0.985 mmol) was dissolved in DCM (10 mL) and cooled in an ice bath. Chlorambucil (200 mg, 0.657 mmol), DMAP (10 mg, 0.065 mmol), and DCC (1 mL, 0.985 mmol) were added to the cold solution, the mixture was purged with argon and stirred in a closed system for 24 h at room temperature. The final mixture was concentrated and purified by column chromatography on silica gel, using EtOAc/Hex up to 10% to extract the title product in 100% yield (228 mg). ^1^H NMR (400 MHz, CDCl_3_): δ 7.06–7.08 (d, *J* = 8, 2H), 6.63–6.65 (d, *J* = 8, 2H), 4.67 (s, 2H), 3.68–3.72(m, 4H), 3.61–3.64 (m, 4H), 2.55–2.59 (t, *J* = 8, 2H), 2.35–2.39 (t, *J* = 8, 2H), 2.47 (t, *J* = 4, 1H), 1.91–1.95 (p, *J* = 8, 2H). MS (M + H^+^): 342.1.

4-(Azidomethyl)-7-((((2*R*,3*S*,4*S*,5*R*)-3,4-dihydroxy-5-(hydroxymethyl)tetrahydrofuran-2-yl)methyl)amino)-2*H*-chromen-2-one (14) [22]: To the solution of 12 (2.3 g, 11.0 mmol) in dry DMF (30 mL) under argon, NaN_3_ (2.85 g, 43.8 mmol) was added, and the mixture was stirred at room temperature for 4 h. The mixture was diluted with EtOAc (400 mL), washed with brine (4 × 100 mL), and dried with MgSO_4_. After filtration and concentration, the residue was absorbed on silica gel and purified by column chromatography on silica gel, eluting with 0−10% methanol in CH_2_Cl_2_. The resulting solid was suspended in CH_2_Cl_2_, followed by addition of hexanes and filtered, washing with hexanes, to provide the title compound as a dark yellow solid in 78% yield (1.86 g). Prolonging the reaction time was found to be detrimental for yields. ^1^H NMR (400 MHz, DMSO-d_6_): *δ*, 7.37–7.35 (d, *J* = 8.4, 1H), 6.58–6.55 (dd, *J_1_* = 2.0, *J_2_* = 8.8, 1H), 6.43 (d, *J* = 2.0, 1H), 6.23 (bs, 2H), 6.05 (s, 1H), 4.69 (s, 2H) ppm. HRMS (ESI): m/z [M + H]^+^ calc’d for C_10_H_9_N_4_O_2_: 217.07257; found: 217.07222.

(1-((7-((((2*R*,3*S*,4*S*,5*R*)-3,4-dihydroxy-5-(hydroxymethyl)tetrahydrofuran-2-yl)methyl)amino)-2-oxo-2*H*-chromen-4-yl)methyl)-1*H*-1,2,3-triazol-4-yl)methyl4-(4-(bis(2-chloroethyl)amino)phenyl)butanoate (**IV**, Man–Click–CLB) [28]: The salt **14** (105 mg, 0.249 mmol) was dissolved in acetonitrile/water 1:1 mixture (*v*/*v*, up to 4 mL) and the solution was purged with argon for few minutes. Propargyl ester (**13**) (114 mg, 0.274 mmol) was added, followed by CuI (75 mg, 0.373 mmol), sodium L-ascorbate (750 mg, 3.735 mmol), and DIEA (129 mg, 3.735 mmol). The headspace of the vial was purged with argon, and the resulting suspension was stirred vigorously at room temperature for 1 h. The product mixture was extracted by ethyl acetate (3 × 10 mL) dried over MgSO_4_, filtered and concentrated under vacuum. The mixtures were then concentrated to dryness under reduced pressure, absorbed on silica, and purified by column chromatography on silica gel using 0−40% methanol in CH_2_Cl_2_ mixtures. Compound **IV** was obtained in 72% yield. HRMS, ^1^H NMR (400 MHz, CD_3_OD): δ 8.11 (s, 1H), 7.48–7.50 (d, *J* = 8, 1H), 6.96–6.98 (d, *J* = 8, 2H), 6.63–6.65 (d, *J* = 8, 2H), 6.50–6.51 (d, *J* = 8, 1H), 5.80 (s, 1H), 5.47 (s, 1H), 5.18 (m, 4H), 4.83 (m, 3H), 3.97–3.98 (m, 1H), 3.91–3.92 (m, 1H), 3.85 (m, 1H), 3.68–3.69 (m, 4H), 3.62–3.63 (m, 4H), 3.40–3.44 (m, 1H), 3.28–3.33 (m, 2H), 2.44 (m, 2H), 2.29 (m, 2H), 1.82 (m, 2H), 1.27 (m, 2H). HRMS (ESI): m/z [M + H]^+^ calc’d for C_10_H_9_N_4_O_2_: 703.21757; found: 703.21744.

### 4.3. Fluorescence Analysis

Analysis of GLUT5 uptake with ManCou-H probe and probes III and IV: Cells were grown in their respective media, plated (300,000/plate) in 35 mm glass-bottom confocal dishes (MatTek) and allowed to grow in their respective growth media for 24 h. For treatment, cell media was removed and 25 µM probe solution in complete media (1 mL) was added. Cells were incubated with probes at 37 °C for 10 min. After incubation, probe solution was removed, and cells were washed with warmed PBS (3 × 1 mL) and leaving 1 mL of PBS for imaging. Cell images were taken using Olympus FluoViewTM FV1000 using the FluoView software. 60× oil suspended lens was used to observe fluorescent activity under DAPI (excitation 405 nm (45% intensity), emission 450/490 nm (30% intensity), 10 µs/pixel.

Analysis of GLUT5 membrane expression through immunofluorescence: Cells were grown in their respective media, plated (300,000/plate) in 35 mm glass-bottom confocal dishes (MatTek), and allowed to grow in their respective growth media for 24 h. At the end of 24 h, the cells were fixed with 4% PFA for 20 min followed by PBS washes (3 × 2 mL) for a total of 15 min. The cells were then protein blocked using bovine serum albumin followed by incubation with primary antibody at a dilution of 1:200 overnight at 4 °C. The samples were further incubated with secondary antibody for 2 h at a dilution of 1:1000 and fluorescence was imaged using Olympus FluoView FV1000 confocal microscope. 60× oil suspended lens was used to observe fluorescent activity with Alexa488 filter.

Corrected total cell fluorescence (CTCF) was derived for every sample after fluorescence quantification with ImageJ. CTCF = Integrated Density − (Area of selected cell × Mean fluorescence of background readings).

### 4.4. Competitive Inhibition Assay

Cells were grown in their respective media, plated (300,000/plate) in 35 mm glass-bottom confocal dishes (MatTek) and allowed to grow in their respective growth media for 24 h. Separate dishes were incubated with the following: 5 µM ManCou-H solution in a complete growth media, a solution of 5 µM ManCou-H and 500 µM fructose in a complete growth media, a solution of 5 µM ManCou-H and 500 µM probe I in a complete growth media, and a solution of 5 µM ManCou-H and 500 µM probe II in a complete growth media. Cells were incubated with probes at 37 °C for 10 min. After incubation, treatment solution was removed, and cells were washed with warmed PBS (3 × 1 mL) and leaving 1 mL of PBS for imaging. Cell images (fluorescence and bright field) were taken using Olympus FluoViewTM FV1000 using the FluoView software. 60X oil suspended lens was used to observe fluorescent activity under DAPI (excitation 405 nm (45% intensity), emission 450/490 nm (30% intensity), 10 µs/pixel. Experiments were carried out in duplicates.

### 4.5. Cell Viability Assay

For biological studies, compounds I–IV were purified by reverse-phase HPLC to achieve 99.9% analytical purity using ACN:Water. 20–70% ACN gradient for I and II and 40–90% ACN gradient was used for III and IV.

For microplate assays, cells were seeded in 96-well flat bottom plates (10,000 cells/well) in 150 µL of culture medium and allowed to grow for 24 h. Cells were then treated with probes (concentration varies) in Media and incubated at 37 °C and 5% CO_2_ for 24, 48, 72 h. After each incubation time, 20 µL of MTS reagent were added and then incubated for 2 and half hours again. According to the MTS suggested protocol, the absorbance of the data was immediately collected using automated UV 96-well plate reader at 490 nm wavelength.

Cell viability was calculated as a relative decrease in the absorbance with respect to the untreated control: Viability, % = (ATreatment − AControl) × 100 (where, A = absorbance). Dose response curves are plotted using Viability, % over log10 of a concentration. The initial zero-point on the x-axis corresponds to all 1 µM concentration treatment. The IC_50_ values, were calculated from dose response curves using Quest Graph™ IC_50_ Calculator, the updated AAT-Bioquest^®^ online calculator tool (AAT Bioquest, Inc., https://www.aatbio.com/tools/ic50-calculator, accessed on 28 April 2021).

### 4.6. Determination of Water Solubility via Octanol–Water Buffer Partitioning

All samples were guaranteed to be of the same weight. Octanol–water buffer partition was performed according to an OECD Guideline for the Testing of Chemicals with a modification using equimolar amounts of compounds [29]. A 4 mL portion of 20 mM pH 7.4 PBS and 4 mL *n*-octanol were introduced into a 10 mL centrifuge tube and mixed to ensure equilibration of the PBS and octanol on a shaker for 24 h. Samples were introduced in equimolar amounts and vortexed for 3 min. After the vortexing, the tubes were centrifuged for 5 min with the rotation speed at 3000 rpm. The lower water layer was then separated, and UV-vis absorbance was recorded.

## 5. Conclusions

Our studies represent the first proof-of-concept of specific targeting of one disease-relevant sugar transporter with bioactive conjugates. We have shown that targeted delivery of a bioactive cargo through one cancer-relevant transporter may prove efficient in inducing cancer-specific cytotoxic response. Through structure-activity analysis, we have shown that the outcomes of specific delivery depend on the ability of the conjugate to maintain GLUT5-mediated passage long-term and that this passage is contingent upon the overall hydrophilicity of the conjugate. The outcomes of these studies provide additional insight into the overall requirements towards bioconjugates for specific delivery through GLUT transporters.

## Figures and Tables

**Table 1:** Cytotoxicity of CLB and conjugates I–IV, IC_50_ (µM) *.

	MCF7	MDA-MB-231	184B5
24 h incubation			
**CLB**	145.2 ± 28.7	155.9 ± 22.3	182.5 ± 33.7
**I**	179.4 ± 24.3	89.9 ± 17.4	>900
**II**	187.6 ± 34.3	194.2 ± 21.9	200/6 ± 18.2
**III**	490.9 ± 21.8	108.4 ± 29.9	203.2 ± 33.2
**IV**	196.8 ± 11.8	>500	200.4 ± 18.4
48 h incubation			
**CLB**	89.7 ± 12.6	114.1 ± 14.4	270.4 ± 24.2
**I**	85.9 ± 27.1	83.6 ± 18.3	>900
**II**	103.2 ± 19.3	99.3 ± 11.1	380.5 ± 23.7
**III**	175.3 ± 10.8	104.1 ± 11.7	241.5 ± 13.2
**IV**	137.6 ± 8.8	108.1 ± 15.3	350.7 ± 33.5

* IC_50_ measured over varied concentrations of bioactive compound in 96-well format using MTS assay. Data are summarized for *n* = 3 independent experiments. The IC_50_ values, were calculated from dose response curves using Quest Graph™ IC_50_ Calculator (AAT Bioquest, Inc., https://www.aatbio.com/tools/ic50-calculator, accessed on 28 April 2021).
